# Localized Lipid Packing of Transmembrane Domains Impedes Integrin Clustering

**DOI:** 10.1371/journal.pcbi.1002948

**Published:** 2013-03-14

**Authors:** Mehrdad Mehrbod, Mohammad R. K. Mofrad

**Affiliations:** 1 Molecular Cell Biomechanics Laboratory, Department of Bioengineering, University of California, Berkeley, California, United States of America; 2 Physical Biosciences Division, Lawrence Berkeley National Laboratory, Berkeley, California, United States of America; University of California San Diego, United States of America

## Abstract

Integrin clustering plays a pivotal role in a host of cell functions. Hetero-dimeric integrin adhesion receptors regulate cell migration, survival, and differentiation by communicating signals bidirectionally across the plasma membrane. Thus far, crystallographic structures of integrin components are solved only separately, and for some integrin types. Also, the sequence of interactions that leads to signal transduction remains ambiguous. Particularly, it remains controversial whether the homo-dimerization of integrin transmembrane domains occurs following the integrin activation (i.e. when integrin ectodomain is stretched out) or if it regulates integrin clustering. This study employs molecular dynamics modeling approaches to address these questions in molecular details and sheds light on the crucial effect of the plasma membrane. Conducting a normal mode analysis of the intact αllbβ3 integrin, it is demonstrated that the ectodomain and transmembrane-cytoplasmic domains are connected via a membrane-proximal hinge region, thus merely transmembrane-cytoplasmic domains are modeled. By measuring the free energy change and force required to form integrin homo-oligomers, this study suggests that the β-subunit homo-oligomerization potentially regulates integrin clustering, as opposed to α-subunit, which appears to be a poor regulator for the clustering process. If α-subunits are to regulate the clustering they should overcome a high-energy barrier formed by a stable lipid pack around them. Finally, an outside-in activation-clustering scenario is speculated, explaining how further loading the already-active integrin affects its homo-oligomerization so that focal adhesions grow in size.

## Author Summary

Focal adhesions are complex, dynamic structures of multiple proteins that act as the cell's mechanical anchorage to its surrounding. Integrins are proteins linking the cell inner and outer environments, which act as a bridge that crosses the cell membrane. Integrins respond to mechanical loads exerted to them by changing their conformations. Several diseases, such as atherosclerosis and different types of cancer, are caused by altered function of integrins. Essential to the formation of focal adhesions is the process of integrin clustering. Bidirectional integrin signaling involves conformational changes in this protein, clustering, and finally the assembly of a large intracellular adhesion complex. Integrin clustering is defined as the interaction of integrins to form lateral assemblies that eventually lead to focal adhesion formation. The effect of the plasma membrane on formation of integrin clusters has been largely neglected in current literature; subsequently some apparently contradictory data has been reported by a number of researchers in the field. Using a molecular dynamics modeling approach, a computational method that simulates systems in a full-atomic scale, we probe the role of the plasma membrane in integrin clustering and hypothesize a clustering scenario that explains the relationship between integrin activation and focal adhesion growth.

## Introduction

Focal adhesions are complex, dynamic structures composed of several proteins that act as the cell mechanical anchorage to the extracellular matrix (ECM). Integrins are the first signal receptors encountered in the cell mechanical micro-environment [1], [2], [3]. Integrin-mediated adhesion often occurs under forces such as fluid flow or myosin-mediated contractions that cells exert to sample the rigidity of their surroundings [4]. The surface density of integrins on the plasma membrane is ∼300 integrins/µm^2^ [5]. The lifetime of focal adhesions as distinct entities is in the order of 5–10 minutes. Within focal adhesions, integrins that are directly linked to the ECM show exchange rates on the order of 1–3 minutes [6]. The exchange rate is defined as the average time it takes for 50% of integrins in a focal adhesion to dissociate from the ECM and replace with new integrins [7].

Integrins are α-β hetero-dimeric receptors that consist of large extracellular domains (700–1000 residues), two transmembrane α-helices, and short cytosolic tails (50–70 residues) [2], [8], [9], [10]. Integrins transfer signals bidirectionally between the ECM and the cytoskeleton [6], [11], [12]. Signal transmission from the cell exterior to the cytoskeleton is called ‘outside-in signaling’, whereas ‘inside-out signaling’ occurs when a biochemical signal is relayed from the cytoskeleton, being converted afterward to a conformational change of the protein. Inside-out signaling is putatively triggered by separation of the two integrin subunits at their cytoplasmic and transmembrane domains, which follows association of talin to the integrin cytoplasmic β-subunit domain [12], [13]. A major restraint that holds integrins in an inactive mode is the interaction between transmembrane and cytoplasmic domains of the α- and β-subunits. Separation of these domains is sufficient to allow unbending of the ligand-binding headpiece and conformational changes that increase ligand-binding affinity [14], [15]. Conversely, binding of the ligand to the integrin ectodomain results in an extended conformation whereby the α- and β-subunit legs are separated [12], [15], [16].

Bidirectional integrin signaling involves conformational changes in the hetero-dimer, integrin clustering, and the assembly of a large intracellular adhesion complex [9], [17], [18]. Integrin clustering is defined as the interaction of hetero-dimers to shape lateral assemblies that eventually lead to focal complex formation [13], [15]. It has been known for two decades that integrin activity is regulated by its conformational changes [11], [19]. Although there is a strong correlation between integrin clustering and activation, how one leads to the other has remained elusive [8], [13], [18], [20], [21], [22]. A body of evidence has proposed that αIIbβ3 integrin activation, which triggers the clustering process, is regulated by α- and β-subunit homo-oligomerization [18], [20], [21], [22]. In opposition to this hypothesis, a series of Cys mutagenesis scanning experiments has suggested that neither inactive nor ligand-bound αIIbβ3 integrins form homomeric association [8]. Instead, it has been hypothesized that integrin clustering occurs as a result of binding of several integrins to multimeric ligands, and the transmembrane-cytoplasmic (TMC) domains do not play any significant role in the process [8]. Additionally, there is evidence that demonstrates it is hetero-dimerization of different integrin molecules that triggers the clustering phenomenon [8], [23].

Studies indicate that focal adhesions alter their size as a result of changes in the force they sustain [11], [24], a process termed “reinforcement” [25]. It has been reported that for focal adhesions larger than 1 µm^2^, traction force at the focal adhesion increases with its size linearly [24]. Current activation/clustering models do not provide an explanation for the force-recruitment correlation in integrins. In contrast, the switchblade model, which is a widely accepted functional model for integrin, limits the integrin's molecular conformations to three modes, namely passive, active, and ligand-bound [19], [26], [27]. The passive mode is corresponding to a bent integrin structure while integrin activation is marked by a global conformational change, which results in a stretched structure with a separation of α- and β transmembrane-cytoplasmic domains from each other. Finally, an opening between the α- and β-subunit heads gives rise to a ligand-bound conformation [26], [27]. Once the ligand-bound conformation is achieved, it is unclear how the reinforcement phenomenon takes place under larger loads.

The effect of the plasma membrane on integrin activation/clustering is largely neglected in previous experimental works, whereas molecular-level, computational studies of integrin are mostly focused on simulating the ectodomain [6], [11], [16]. In this study, we employ all-atom molecular dynamics techniques and conclude that the plasma membrane surrounding the integrin transmembrane domains plays an important role in the process of activation/clustering by forming a lipid pack around the transmembrane domains. Furthermore, by performing normal mode analysis on the full-length αIIbβ3 molecule, we show that the integrin ectodomain and TMC domains swing about a hinge-like region that links them together. Finally, we hypothesize a potential integrin clustering scenario that explains the seemingly contradictory results for integrin clustering mechanism, namely homo-oligomerization-based and multivalent ligand-based clustering models, elucidating the unexplained force-area correlation in focal adhesions as well. One of the major determining factors for feasibility of a reaction is the free energy change profile over the reaction coordinate. Therefore, we finally asked if integrin subunits were to oligomerize how the free energy of the system would change as the two monomers approached one another.

## Results

### Normal mode analysis of full integrin

**Figure 1:**
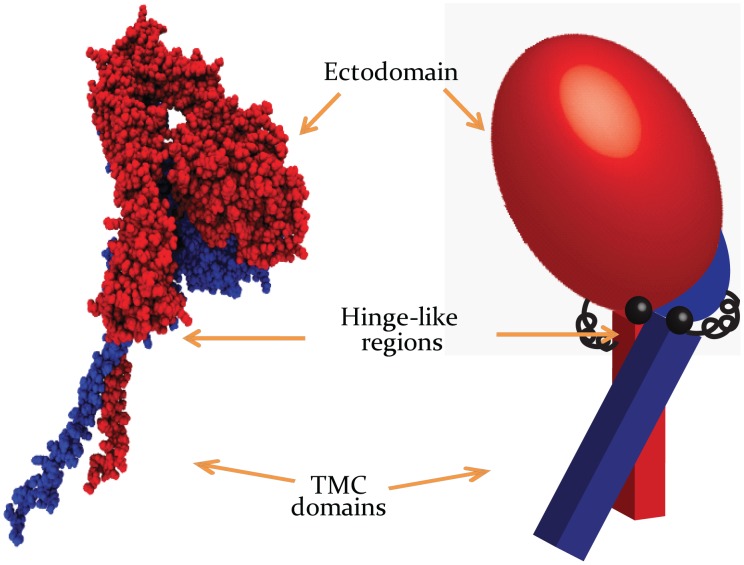
A simplistic, mechanical equivalent for αIIbβ3 integrin. (Left) full-length integrin αIIbβ3A dimer consists of an α-subunit (red) and a β-subunit (blue). (Right) A purely mechanical model is suggested, illustrating how TMC domains are hinged to the more rigid ectodomain while rotational springs represent the weak bending stiffness at joints. See also Figures S1 and S2.

Normal mode analysis is a powerful method to detect softer regions in proteins and has proved useful in determining physiologically relevant motions of proteins as well as their compartmental breakpoints [28], [29], [30], [31]. Our normal mode analysis of the full-length integrin αIIbβ3 suggested that the protein's structure consists of a rather rigid ectodomain, hinged to two cantilever-like transmembrane-cytoplasmic (TMC) domains (see Fig. 1). Modes 7 to 10, corresponding to lowest non-zero deformation energies, show a clear rotation of the ectodomain and TMC domains about the hinge-like region (see Fig. S1). The soft linker region between the ectodomain and TMC domains hinders strong mechanical integrity of TMC domains with the extracellular domain. As expected, under an instantaneous excitation the ectodomain and TMC domains vibrate about the soft region in opposite directions at any given time. As mode number increases the molecule deformation energy is elevated rapidly, and consequently, contribution of higher modes to the molecule movement is insignificant (Fig. S2) [32]. Additionally, no homomeric interactions are reported to occur between integrin ectodomains [8]. Therefore, in order to reduce the computational cost of our simulations, in all molecular dynamics simulations solely the TMC α- and/or β-domains were modeled.

### Integrin α-α homo-oligomerization

**Figure 2:**
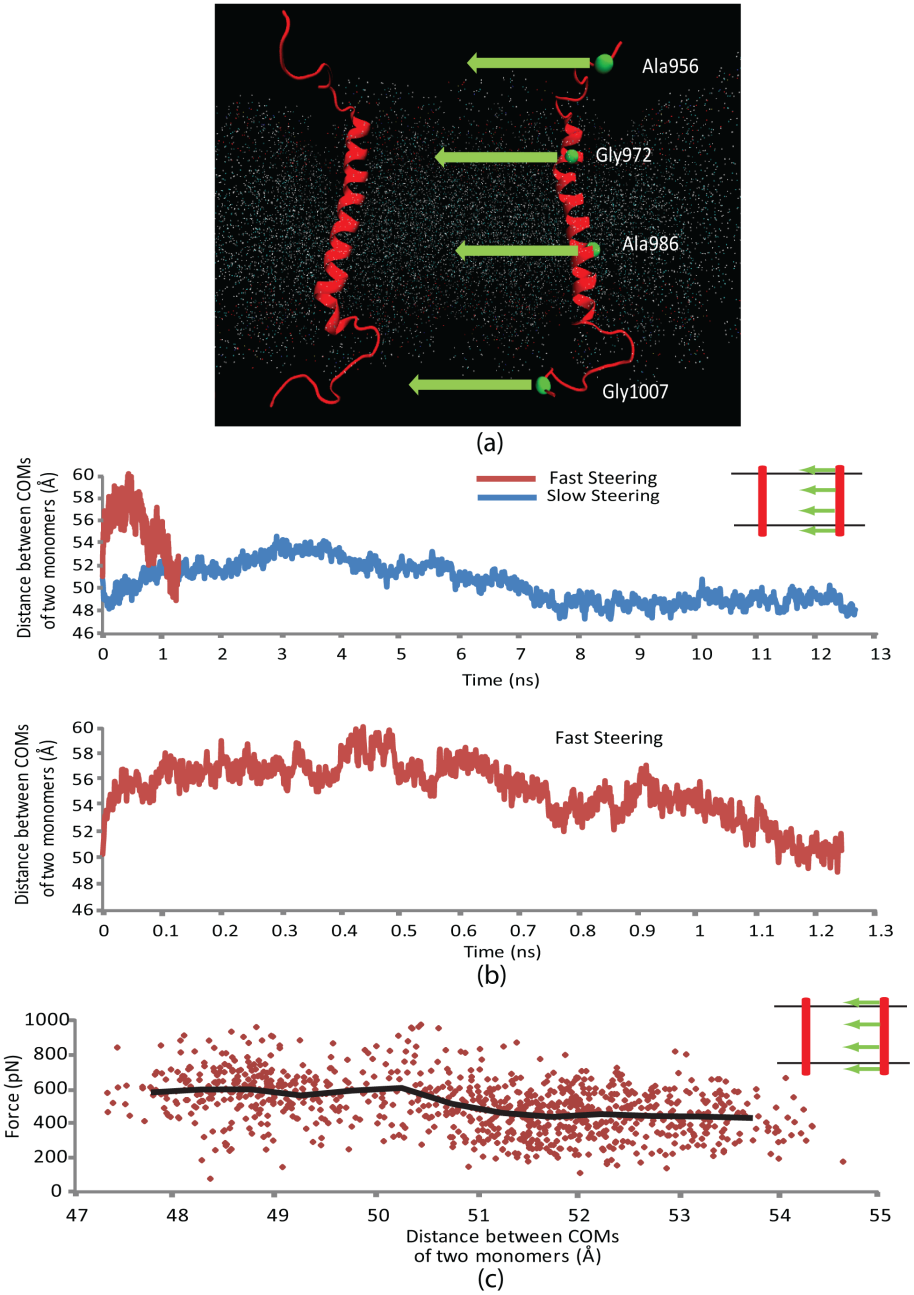
Interaction between two transmembrane α-subunits when one is steered and the other is free. (a) Two transmembrane-cytoplasmic domains of integrin α-subunit are embedded in a lipid bilayer patch (shown by scattered points). The green spheres represent the steered atoms being pulled along the green arrows. (b) Distance between centers of mass of the two α-subunit monomers as a function of time for two different pulling rates. The slow steering velocity is 0.0025 Å/ps whereas the fast steering velocity is 0.025 Å/ps. The diagram legend shows how one α-monomer (red) is pushed toward the other free one while both are embedded in a lipid bilayer. Diagrams for the two steering speeds are juxtaposed in the upper diagram to show that the final distance between the monomers is not dependent upon steering speed. The lower diagram provides more details for the slow speed diagram. (c) The pulling force needed to steer one monomer toward the other is plotted. When monomers are more than 5.1 nm apart, about 400 pN is required to drag the molecule. This ramps up as the monomers further approach toward each other, and eventually levels off when the final distance is attained. The heavy black line is a window-averaged form of the data with the window size of 0.5 nm.

The first step to our molecular dynamics simulations was aimed at investigating the homomeric interactions between the two α-subunits when they are in an active state (i.e. α- and β-subunits are distant enough not to interact significantly). In order for integrin α-subunits to initiate focal adhesion formation through their homo-oligomerization, α-subunits should be able to readily bind/unbind each other. Two identical crystal structures of integrin αIIbβ3 TMC α-subunits (Gly955 to Glu1008) were carefully embedded in a plasma membrane patch, 5 nm apart from one another. The system was minimized and equilibrated for 0.5 ns and steered molecular dynamics (SMD) was exploited to drag one of the α-subunits toward the other for 1.2 ns with a constant velocity of 0.025 Å/ps, while the other monomer was left unconstrained. Importantly, the free monomer also started to move in the same direction soon after the steered one did. Furthermore, the overall force required to direct the steered monomer toward the free subunit showed an abrupt increase at distance 5.1 nm (see Fig. 2c), and electrostatic as well as van der Waals energies of the interaction between the two monomers were insignificant during a major part of the simulation (data not shown). This indicated that the two biomolecules did not ‘see’ each other throughout the simulation. Some minor positive interaction energy spikes were observed, which, based on the trajectory of the simulation, can be attributed to interactions between a GAMG sequence located at the very end of the extracellular side of the steered α-subunit, where it is truncated off the ectodomain (Fig. S3). Conducting the same simulation but without the GAMG motif demonstrated almost no interaction energy at any time (see Fig. S3). This negative control suggests that the emerging energy spikes were an artifact of the separation of the integrin transmembrane and extracellular domains that had left this flexible linker region unconstrained, allowing the GAMG sequence to reach out to the other monomer. Another site of interaction was the hydrophobic patch on the cytoplasmic side of the free monomer, which also extended out from the plasma membrane. We further explored the phenomenon by repeating the simulation using a geometry in which the steered monomer's orientation is 90° rotated about its longitudinal axis relative to the free molecule. This time the free monomer maintained a distance of ∼4 nm (data not shown).

To observe if the pulling rate has a significant effect on results, the simulation was performed for a longer time scale with a pulling velocity as low as 0.0025 Å/ps. The results were, in general, similar to the first simulation except that the fluctuations of the distance between the monomers fell in a narrower band. Nevertheless, as Fig. 2b depicts, the distance in both cases eventually approaches the same value (5–5.1 nm), which is the closest distance between centers of mass that the two monomers can reach without any constraints applied. We call this distance “final distance” hereinafter. The increase in the force level required to maintain the constant pulling rate also occurs at the distance of 5 to 5.1 nm.

**Figure 3:**
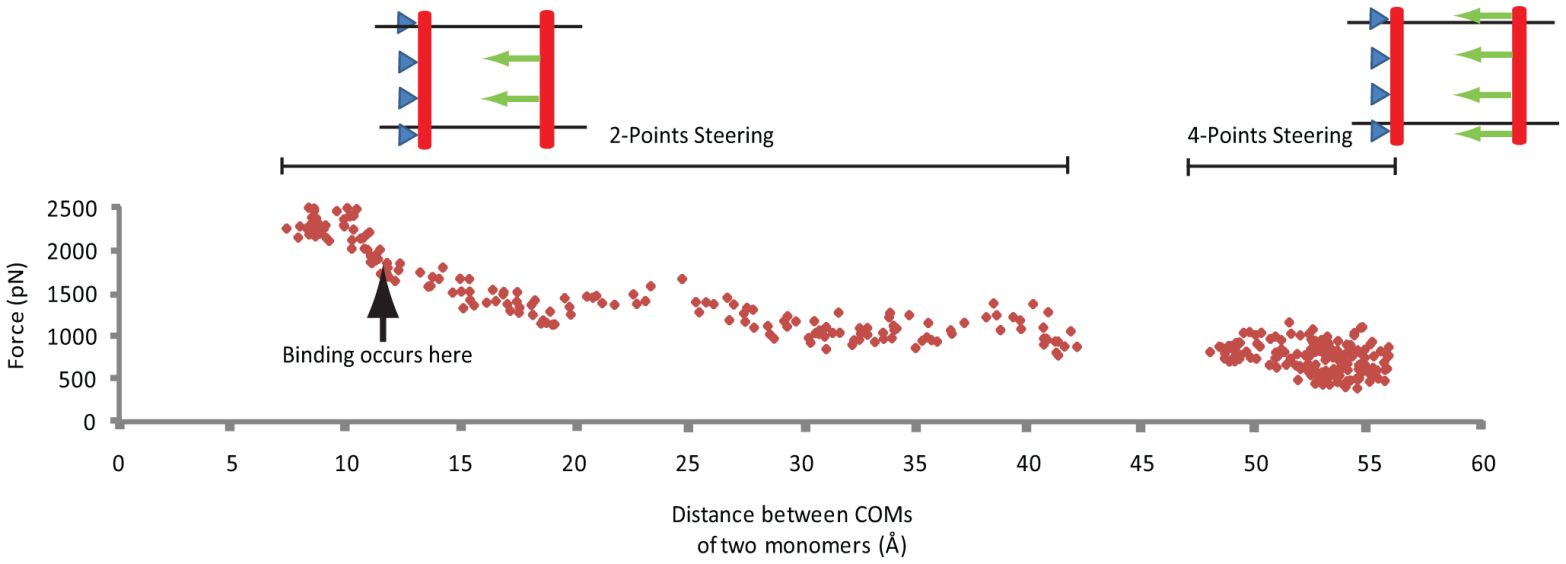
Molecular dynamics results of two α-monomers when one is fixed and the other is steered. Results of SMD simulation of one α-monomer as the other is constrained. The force required to drag the monomer rises gradually as the distance decreases, when the monomers are further than 5.1 nm apart from one another. Results of the 4-points and 2-points SMD are juxtaposed.

Mimicking α-dimerization appeared unlikely if α-subunits are not constrained. Thus in the next round of simulations we fixed the non-steered monomer at the same four atoms (Cα of residues Ala956, Gly972, Ala 986, and Gly1007). The steered monomer was pulled on toward the unconstrained one at the same four atoms as before, which were selected such that they divide the monomer into similar length spans, and fixed atoms with smallest side chains are chosen to reduce the potential error caused by steering. The steered monomer approaching the constrained one, the pulling force rose again when the monomers were ∼5.1 nm apart. The four-point SMD was deemed incapable of mimicking the full dimerization process. The pulling mechanism used in our simulations assumed a dummy point that moved in the space with a constant velocity and pulled the steered points on the molecule, using dummy springs that are linking those points to the dummy point. In fact, when the α-monomer was being pulled from four points, the two points at the two ends of the monomer fell inside the water box while the two points in the middle were embedded in the lipid bilayer. As a result, when the lipid packs around the two monomers started to overlap, the two embedded points experienced a substantially higher resistance than the ones moving in the water box, and therefore fell behind the lipid embedded points. This initially led to unfolding of the steered monomer. We avoided this problem by releasing the two end points when the steered monomer approached the fixed one. Hence, the simulation was continued one more time with only two, membrane-embedded steering residues (i.e. Gly976 and Ala986) (Fig. 3). These two complementary simulations, therefore, conferred a profile of the overall force required to tow a single TMC α-domain toward the other constrained one. An overall SMD force of as much as 1.7 nN is required to eventually bind the two α-domains.

### Lipid packing phenomena

Observing the system trajectory from the top view (perpendicular to the membrane patch), it appeared that two cylinder-like batches of lipid chains surrounding the α-subunits moved along with the monomers. As shown in Fig. S4a, although a portion of lipid chains originally located in the proximity of the monomer disperses in different directions, the majority of lipid molecules within at least 10 Å of the transmembrane domain are more energetically favorable to remain attached to the monomer. In order to further quantify the phenomenon, we measured the distance between the monomer's center of mass and three lipid chains located at the rear side of the moving monomer, along the line that connects the two monomers, with different initial distances from the monomer. Intriguingly, the lipid chain that was originally located as close as ∼12 Å to the monomer's center of mass almost kept a constant distance with the monomer, which means it moves with a constant velocity along with the monomer. Nonetheless, the farther the lipid chain located from the monomer, the more slowly the lipid chain was dragged behind the monomer. This is visualized in Fig. S4b by monitoring the distance between the monomer and lipid chain.

**Figure 4:**
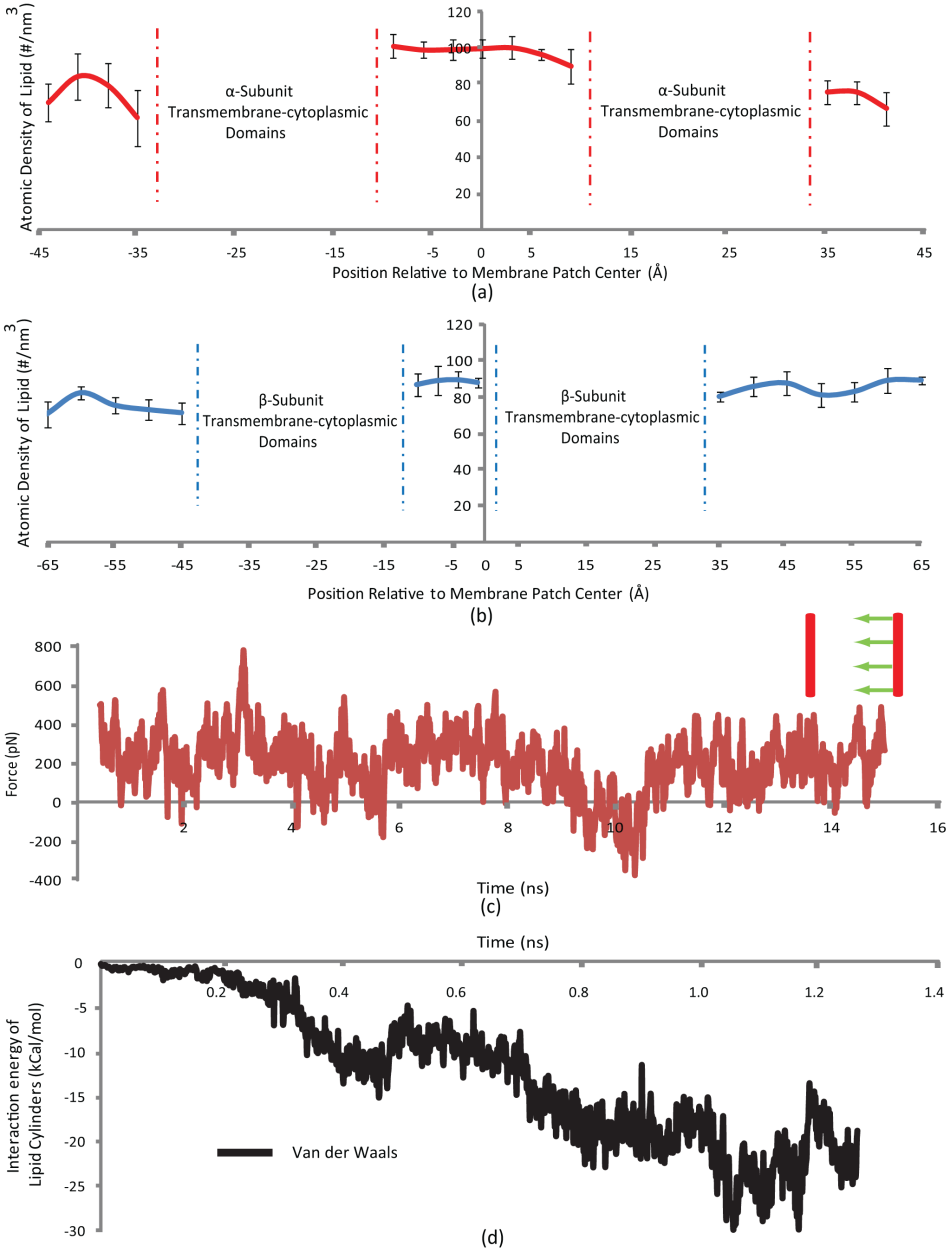
A membrane barrier hinders α-subunit homo-oligomerization. Lipid packing is suggested to have a major effect on integrin homo-oligomerization. (a) The lipid density of the cytoplasmic compartment of the plasma membrane in the area enclosed by the two α-domains is significantly higher than the density of regions outside α-domains by ∼20%. (b) The lipid dense region forms between β-domains too. However, the contrast between the regions defined by the two β-domains with outside is much lower. (c) The steered and free α-monomers readily interact when the membrane is absent. Pulling force remains constant until t = 0.8 ns when the interaction between the two monomers begins. The pulling force reaches a minimum at t = 1 ns when the helix surfaces are in contact and the hydrophobic attraction replaces the required pulling force gradually, until it eventually takes a negative amount. The legend illustrates the steered α-monomer approaching the free one while both move outside the lipid bilayer. (d) van der Waals interaction energy between lipid cylinders around the two α-monomers is a measure of their contact. It takes a maximum at t = 1 ns which is indicative of the full interaction between the lipid cylinders.

It was speculated that “lipid packing” around the monomers might be the underlying reason for why the free monomer was “repelled” from the steered monomer. Thus, the atomic density (number of atoms per volume) of lipid around both α- and β-subunits was measured as a function of the lipid position, relative to the membrane patch center. Interestingly, as depicted in Fig. 4a and 4b, the atomic density of lipid in the region surrounding the two α-subunits is approximately 20% higher than the density of the free areas on the two sides of the membrane patch. However, the lipid arrangement around β-subunits does not show this much of a contrast. Although the density of the lipid between the two β-subunits is also higher than other regions, it appears that formation of a significantly denser lipid chain conformation between α-subunit TMC domains is favorable, which hinders oligomerization. A major portion of the transmembrane domain of α-subunit consists of hydrophobic residues, which makes it favorable for lipid chains to form a denser pack in the region defined by two α-subunits. Although it is challenging to verify this phenomenon experimentally, computational models allowed us to investigate this system in molecular detail.

To verify potential effects of the lipid bilayer, a control simulation was performed exclusively with two TMC α-subunits with no surrounding membrane. Interestingly, this time the repulsion of the free monomer was not observed, rather the two monomers simply approached each other and eventually associated together (see Fig. 4c). The pulling force remained constant until t = 900 ps, when the interaction between the two monomers began. The pulling force reached a negative minimum at t = 1000 ps when the helix surfaces were in contact and the hydrophobic attraction between the monomers was so strong that the steered monomer was effectively pulled back to keep the velocity constant (see Fig. 4c). Subsequently, the steered helix compelled the free one to move along with it, elevating the force back to the level exhibited prior to the interaction. It should be noted that the minimum separation reached by the α-monomers was ∼2 nm. Therefore, the average effective radius of each monomer, considering the steric repulsion caused by side chains, could be estimated to be ∼1 nm.

As mentioned earlier, a portion of the lipid side chains surrounding each monomer traveled along with the α-helix. In the simulation where a TMC α-domain was directed toward another free one, the van der Waals interaction energy between the two lipid cylinders, each with external radius of 2.5 nm, was computed (see Fig. 4d). The final distance in this case was 5.1 nm. As the system is geometrically symmetric relative to the monomers, it could be concluded that a lipid cylinder of 2.55 nm forms around each membrane-embedded α-monomer. Nonetheless, it is noteworthy that the cylinder is not perfect, meaning its radius differs along different directions. Since the effective radius of each α-monomer (considering the steric repulsion caused by side chains) is more than 1 nm the lipid cylinder average radius is estimated to be 1.5 nm. The result indicated a rise in the interaction level between the two semi-cylinders until t = 1000 ps. The interaction energy continued fluctuating about a plateau afterwards. At this moment, the two monomers were at their final distance and the steered monomer pushed off the free one via their lipid cylinders (Fig. 4d).

These results implied that the repulsion is caused by lipid packing around monomers. Given the highly hydrophobic nature of the membrane-embedded residues of the TMC α-integrin, when the two monomers are far enough from each other, local interactions between their surrounding lipid chains and their hydrophobic residues occupy their hydrophobic patches' binding sites, preventing the buried residues in the two monomers from interacting. Furthermore, these hydrophobic interactions form a dense pack of lipid chains around each monomer. Once the two lipid packs overlap, they are forced away due to steric repulsion. The friction of the two lipid packs with their surrounding lipids creates a barrier for each movement step of the pack. Therefore, the free monomer is pushed off intermittently rather than being shifted smoothly. Therefore, if the two monomers are to dimerized, external, mechanical work is needed to overcome the energy barrier that maintains the packed lipid chains together, letting the monomers pass through.

### α-dimerization

**Figure 5:**
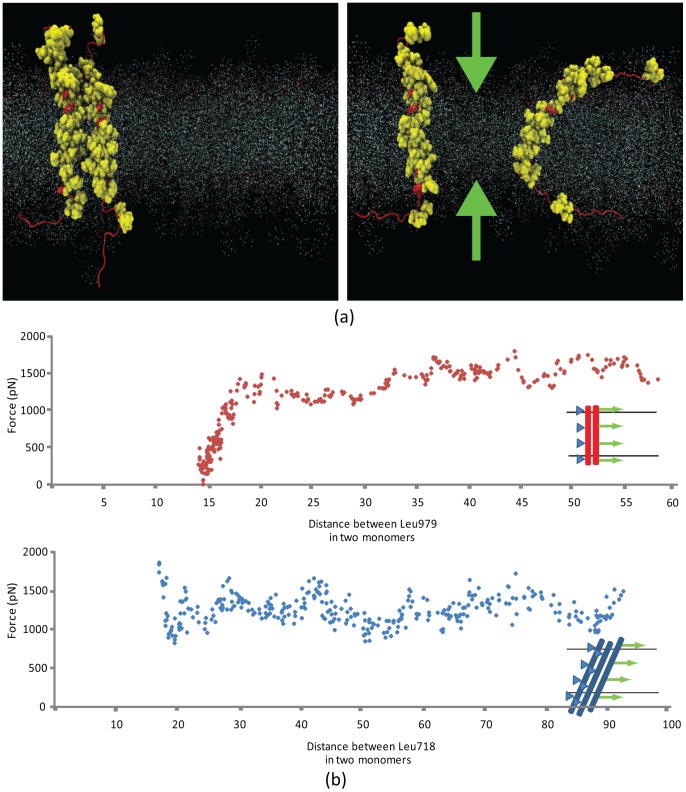
Forced disassembly of integrin nano-clusters. (a) Concomitant with the lipid barrier breakage the plasma membrane thins out in the region between the two α-monomers. (b) Time-force diagrams of α-dimerization (top) and β-trimerization (bottom). The legends show an α-monomer being pulled away from another constrained one, and a β-monomer being steered off of a β-trimer complex.

Proposed by some researchers as an appropriate choice to regulate integrin clustering/activation, the TMC α-domains should be able to associate and detach from one another readily [20], [21]. A dimerization test was carried out in order to characterize the α-domain homo-oligomerization process. Two α-domains were positioned in a plasma membrane patch as close as 2 nm apart. The system was minimized and equilibrated for 2 ns. Interestingly, at this time, the two monomers bonded to each other fairly rapidly (∼50 ps after the simulation started). Afterward, one monomer was fixed (at the same four points), while the other monomer was pulled away with a constant velocity (0.025 Å/ps). The force increased linearly with the steered monomer moving away from the fixed one. A maximum force of 1.6 nN was required to completely separate the monomers. Concomitant with the lipid barrier breakage, the plasma membrane thins out in the region between the two α-monomers (Fig. 5a). This phenomenon again affirms the movement of a significant mass of lipid with the steered monomer.

### Integrin β-β homo-oligomerization

Another crucial element of integrin activation/clustering is β-subunit, which is another candidate reported to be an initiator for integrin clustering and focal adhesion formation consequently. We probed the effect of outside-in signaling on the TMC β-domain by conducting a simulation of two TMC β-domains (Gly684 to Thr762), one constrained at four atoms (Cα of Val695, Gly708, Ala728, and Ala742) while the other one is steered (at the same four points) toward it with a constant velocity of 0.025 Å/ps. Regions of the β-domains falling outside the membrane interacted first, trapping the system in a stable conformation. The stability of the oligomerized state was confirmed by removing the steering force after the dimer was formed and the system was equilibrated for 3 ns. It was observed that the system did not change its conformation significantly after this period of equilibration.

**Figure 6:**
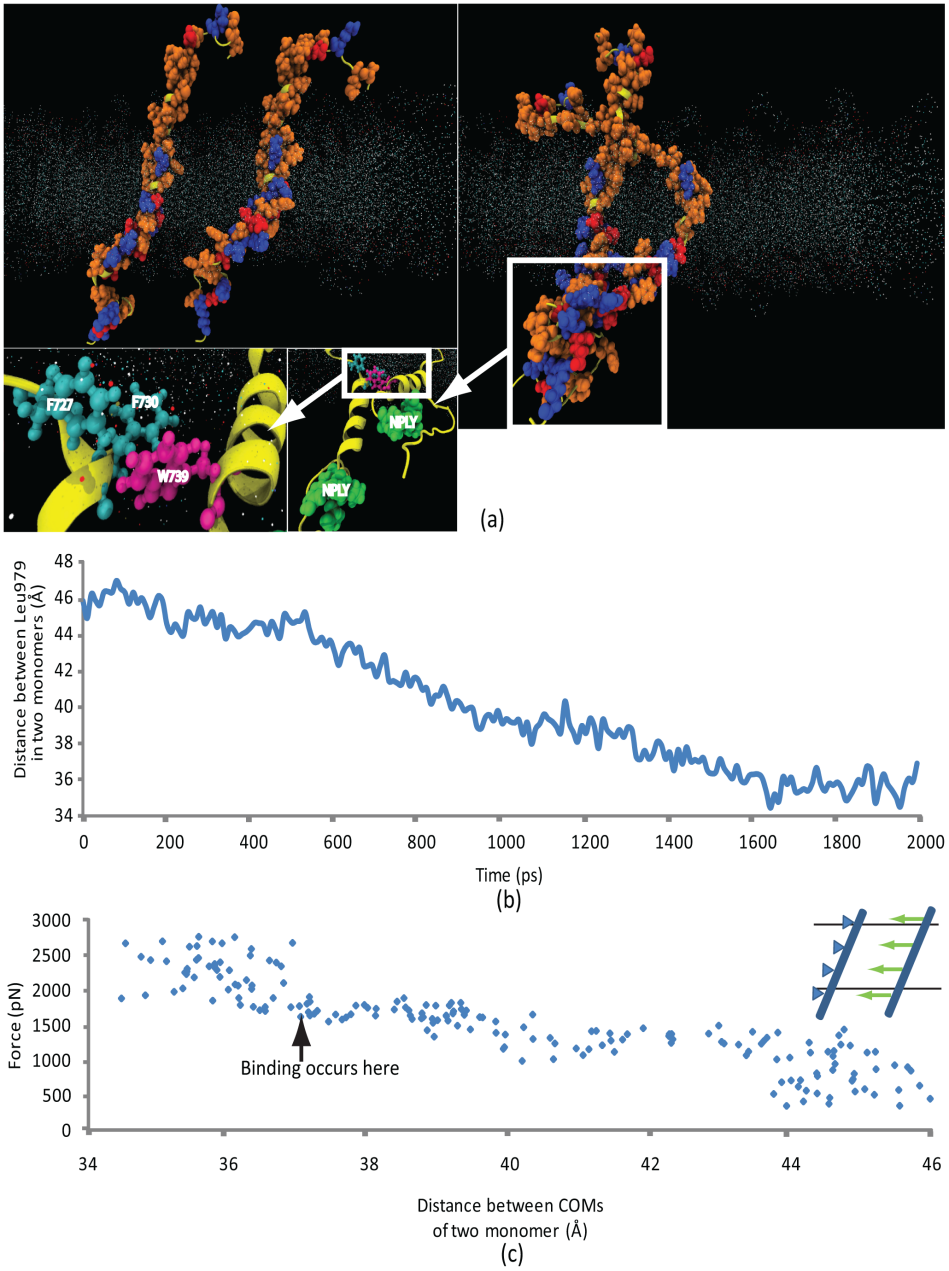
Interaction of two β-subunits when one of them is fixed and the other is steered. (a) (top left) Two β-subunits are placed 5 nm apart from one another (helix backbone: yellow; hydrophobic residues: orange; acidic residues: red; basic residues: blue) and equilibrated in a plasma membrane patch (point representation). Membrane-proximal, cytoplasmic residue W739 on the steered monomer and residues F727 and F730 on the constrained monomer interact with the plasma membrane strongly enough to introduce a kink between the cytoplasmic and transmembrane domains of each monomer. (top right) Steered residue W739, and constrained residues F727 and F730 form hydrophobic bonds, shaping a stable conformation. (inset) The NPLY sequences, where talin-integrin interaction occurs, are highlighted. (b) Distance between the centers of mass of the monomers diminishes linearly until the two monomers interact at distance 3.6 nm. (c) The SMD force increases as the steered monomer comes closer, and the interaction eventually occurs at 1500 pN. The legend illustrates the steered β-monomer (blue), being pulled toward the constrained monomer while they are embedded in a lipid bilayer.

The process of β-dimerization took place before the monomers paired up completely, leaving some lipid chains trapped in between (Fig. 6a). In fact, the lipid packed around the monomers partially ruptured at 1.5 nN and let the β-monomer through. Lower density of hydrophobic residues in the cytoplasm-proximal side of the transmembrane domains forms a region of narrow lipid packing, further facilitating the interaction between the cytoplasmic domains of the β-monomers. Membrane-proximal, cytoplasmic residue W739 on the steered monomer and residues F727 and F730 on the constrained monomer interacted with the plasma membrane strongly enough to introduce a kink between the cytoplasmic and transmembrane domains of each monomer that signifies the partially hydrophilic region of the transmembrane protein within the cytoplasmic leaflet (Fig. 6a). Residue W739 of the steered monomer and residues F727 and F730 of the constrained monomer form hydrophobic bonds, shaping a stable conformation.

The relative position of Cα of Leu718 residues on the two monomers are depicted in Fig. 6a, which represents the geometric center of the lipid-embedded portions of the monomers. The distance drops linearly down to 36 Å and remains constant afterward, at a level close enough to allow the cytoplasmic and extracellular regions of the transmembrane domains to interact.

### β-trimerization

As β-domains are often reported to form trimers in focal adhesions, we placed them on vertices of an equiangular triangle with side length 2 nm. Trimerization occurred quickly within 50 ps. Again, we exploited steered molecular dynamics to separate one of the β-subunits from the trimer, exerting forces on four atoms (Cα of Val695, Gly708, Ala728, and Ala742). The steered monomer separated from other two at a maximum of 1.3 nN. In contrast to TMC α-domains, which have an entirely hydrophilic membrane-embedded compartment, these residues are sparse on the TMC β-domains cytoplasm-proximal compartment (starting from D723). By narrowing the lipid pack barrier, this forms a weak spot where the lipid barrier cuts open in a zipper-like fashion. In other words, the steered β-domain detachment initiates at the cytoplasm-distal region. As the steered molecule is further forced other bonds break one at a time. This appears in Fig. 5, where each peak represents a bond-breaking event.

### Free energy of dimerization

**Figure 7:**
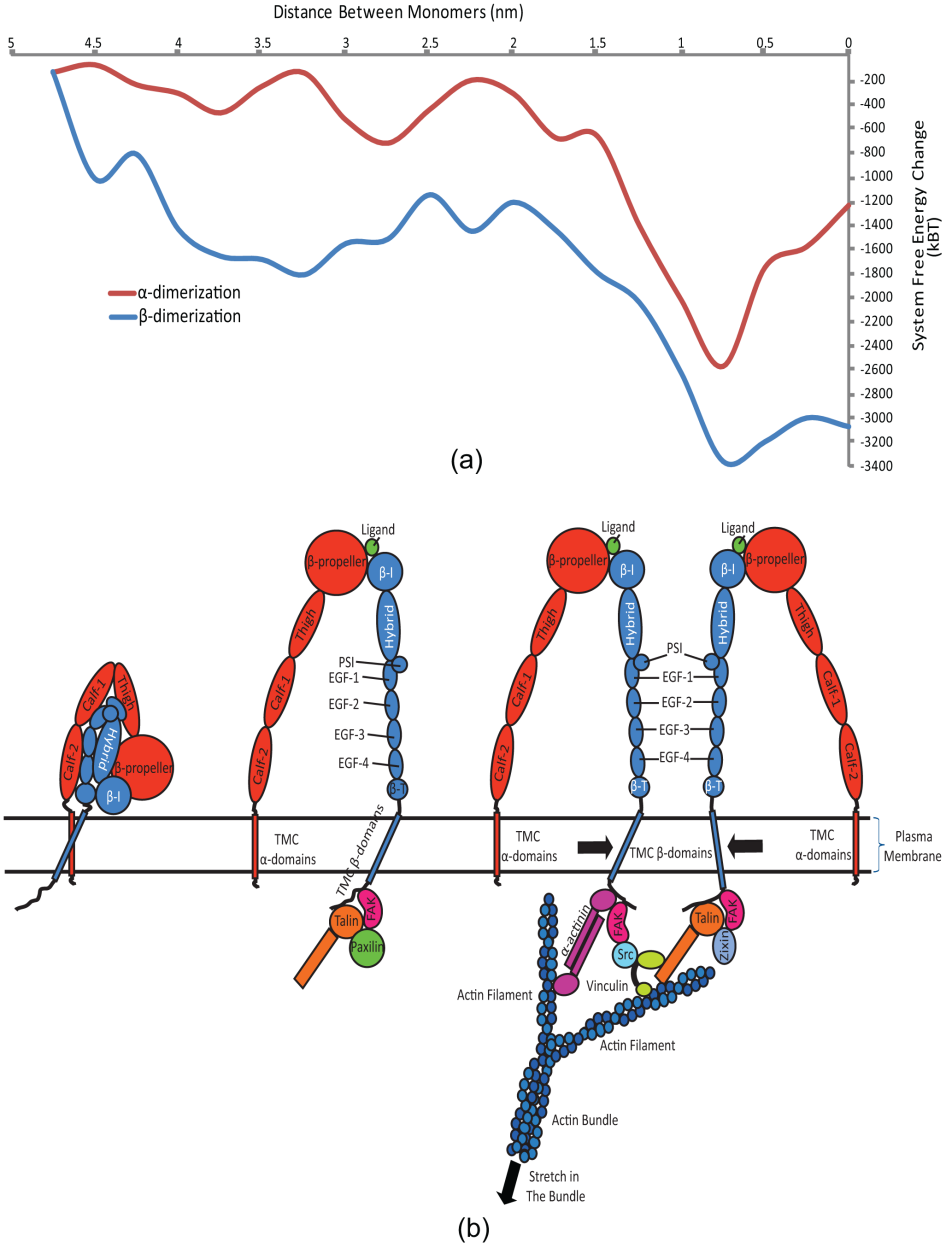
Integrin TMC β-subunits potentially cause integrin clustering and focal adhesion growth through binding with the cytoskeleton. While β-subunit homo-oligomerization could be a candidate mechanism underlying integrin clustering and focal adhesion formation, α-subunits are not as likely to form oligomers. (a) The free energy profiles of α- as well as β-subunit association are plotted against each other. The free energy of the system decreases gradually as either of the moving α-subunit or β-subunit approaches their free counterparts. The effect of the lipid pack disturbance is seen at the few local peaks that appear between 2 ns and 3.5 ns for both subunits, and finally, the free energy drops drastically as a result of the two monomers interacting and forming a dimer. Interestingly enough, the global free energy minimum for the oligomerized state for α- and β-monomers coincides. However, the α-monomer indicates a sharper drop in the free energy with about 10% higher free energy difference between the peak and valley. Furthermore, α-monomers require about twice as much activation energy as do β-monomers to overcome local free energy maxima. (b) A schematic for a potential integrin activation and clustering scenario (i) In an inactive integrin molecule the transmembrane α- and β-domains are tightly bonded. (ii) Once integrin is activated, it forms a micro-cluster of focal complex proteins around it. (iii) After the focal complex matured to a focal adhesion the clusters grow and bind to actin filaments. If the tension in an actin filament is increased, its sub-filaments come closer to one another, bringing their corresponding β-domains together, thereby increasing the focal adhesion area by recruiting more β-integrins in trimers.

In order to further evaluate possibility of integrin subunit homo-oligomerization we analyzed the free energy change of the system as one TMC subunit approached the other for either of α- and β-subunits (see Fig. 7.a). To model the system more accurately, rather than solely bringing the monomers close to each other, we included the effects of the linkage of β-subunits to the cytoskeleton through talin. We started from an initial step where two β-subunits are embedded in the plasma membrane patch, 5 nm apart. Then, we exerted the steering force only on the cytoplasmic domain of the moving monomer (i.e. Ala750-Thr762), while the other monomer was left to diffuse freely. The steering speed was kept constant at 0.025 Å/ps and the two cytoplasmic domains dimerized after 2 ns. The same protocol was employed to measure the free energy difference for α-dimerization.

To calculate the free energy of interaction we invoked the Jarzynski method for non-equilibrium transitions in the system phase space [33]. This method has been able to closely estimate the free energy difference in molecular dynamics simulations by sampling the system for a limited number of times (e.g. 10 times) [34]. To achieve reliable results from the Jarzynski method, sufficient number of identical systems should be minimized and equilibrated separately before they are steered [35]. We minimized and equilibrated the system for either of α- and β-subunit homo-oligomerization profiles 10 times for 0.5 ns, each starting from the same initial state. The systems were steered subsequently and the free energy was evaluated using a second-order expansion of the Jarzynski equality [33], [34]. The free energy profile of the system is shown in Fig. 7a. The free energy profiles of α- as well as β-subunit association are plotted against each other. The free energy of the system decreases gradually as either of the moving α- or β-subunit approaches the free subunit. The effect of the lipid pack disturbance is seen at the few local peaks that appear between 2 nm and 3.5 nm for both subunits, and finally, the free energy drops drastically as a result of the two monomers interacting and forming dimers. Interestingly, the global free energy minimum for the oligomerized states of the α- or β-subunits closely coincide. However, the α-monomer indicates a sharper drop in the free energy with about 10% higher free energy difference between its peak and valley. Furthermore, α-monomers require about twice as much activation energy as do β-monomers to overcome local free energy maxima. The free energy barrier that should be overcome by β-subunits to homo-oligomerize is about 400k_B_T. Although this value is too high for two free β-subunits to overcome, the mechanical work caused by their linkage to the cytoskeleton is likely to push β-subunits over this free energy bump, which would lead to oligomerization. On the other hand, α-subunits would require as high as 800k_B_T of activation energy to overcome the oligomerization energy barrier. Considering that no significant mechanical linkage between α-subunits and the cytoskeleton has been reported, the homo-oligomerization seems thermodynamically unlikely.

## Discussion

Although the role of integrin activation in integrin clustering and vice versa has been studied intensively over the past decade, the phenomenon still remains ambiguous in many aspects [8], [21], [22]. Some researchers suggested that monomer homo-oligomerization of transmembrane integrin α- and β-subunits triggers integrin clustering [21], [22]. However, other researchers provided evidence that homo-oligomerization of integrin subunits does not significantly take part in the process of integrin clustering [8]. Because real-time monitoring of single integrins proved challenging, previous studies utilized assays that involved collecting integrins from cells that are already lysed [8], [20], [21], [22], which implied neglecting effects of the plasma membrane. This study aimed at investigating effects of the plasma membrane on the integrin clustering phenomenon through a full-atom simulation.

Molecular dynamics has been widely recruited to model mechanistic behaviors of cytoskeletal proteins [36]. Particularly, Kalli et al. in their recent work provided evidence that molecular dynamics simulations are able to closely mimic the hetero-dimerization of transmembrane domains of integrin, when embedded in a lipid bilayer [37]. With no experimental techniques currently available to obtain dynamic, atomic-level insights into the integrin activation pathway, we employed steered molecular dynamics (SMD) simulations to derive these insights computationally. Because integrin has a massive structure usually only relevant-to-the-problem portion of integrin is truncated and modeled in molecular dynamics simulations [11], [26]. Also, it is believed that the membrane-proximal segment of the ectodomain is a soft region that links the transmembrane domain and the ectodomain. We conducted a normal mode analysis on the full-length integrin molecule to show the presence of a hinge-like region that minimizes the mechanical integrity of the extracellular and TMC domains of the integrin [2], [9]. Thus, we circumvented the extremely high computational cost of simulating an intact integrin embedded in a membrane, by including only the TMC domains in our simulations.

Inactive integrin α- and β-subunits are locked by two transmembrane interactions: Outer membrane clasp (OMC) and inner membrane clasp (IMC). The GXXXG motifs are thought to be sufficient for keeping the two hetero-dimers in contact [38]. Also these sequences allegedly play a significant role in the process of homo-oligomerization [21]. It has been demonstrated that transmembrane α-domains in an activated integrin tend to form dimers, while transmembrane β-domains are more inclined toward homo-trimerization [20], [21], [22]. Fluorescence microscopy studies demonstrated that there is no interaction between α- and β- cytoplasmic tails in the active state, and they are at least 10 nm apart [39]. Therefore, in all simulations we mimicked the ligand-bound mode by employing non-bonded TMC α- and/or β-domains as opposed to the inactive case where these domains are in a hetero-dimerized state. Ectodomains are associated with the ECM so they could not have a significant effect on homo-oligomerization [8].

In fact, diffusion of transmembrane proteins, including integrins, occurs significantly slower *in vivo* compared to how rapidly it would occur in artificial lipid bilayers. This is suggested to be caused by the physical blocking of their cytoplasmic tails by the cortical cytoskeleton [40]. Lepzelter and Zaman proposed the value of 0.25 nm^2^/µs for the free diffusion coefficient of integrin dimers [40]. Assuming a 2D random walk on the membrane, we can employ the Green's function of diffusion to correlate the integrin position variance from the movement origin with the diffusion time as follows:
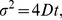
(1)where *σ^2^*, *D*, and *t* are respectively the position variance, diffusion coefficient, and diffusing time. Assuming a normal distribution, a single integrin traveling from the origin will fall within 3σ of the origin at all times with 99.7% certainty. The time scale is 2 ns for most of our simulations. Substituting these values in Eq. 1 yields a standard deviation of 0.0447 nm, consequently, 3σ = 0.1342 nm. Dividing this by the time scale of 2 ns gives a characteristic velocity of 0.0671 nm/ns. However, this is the molecule average speed from its hypothetical origin, rather than its instantaneous speed at each walking time step, which is what is “mechanically sensed” by its surrounding. Indeed, the instantaneous speed could be much greater than this. According to the central limit theorem, if the molecule takes steps of size Δx each in time span of Δt, variance of the position could be calculated as:
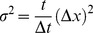
(2)Comparing Eq. 1 and 2, noting that 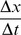 is simply the instantaneous (time step) speed for small time steps, and rearranging, we are left with the following expression for the instantaneous speed:
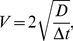
(3)In which we denote the instantaneous speed with *V*. Substituting 0.25 nm^2^/µs and 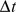 = 2 fs (the time step used in all simulations), the instantaneous speed is V = 22.36 nm/ns. We used a pulling velocity of 2.5 nm/ns, which is an order of magnitude less than the average instantaneous speed in order to avoid sudden sharp movements of the steered monomer that would otherwise impose a significant impulse to the free monomer.

Our simulations showed that a high level of hydrophobicity in the transmembrane α-domain combined with its perpendicular-to-membrane orientation forms a lipid pack of ∼1.5 nm thick around its effective surface. In addition, in the case where two α-subunits are to approach one another, the density of lipid chains was shown to be significantly higher in the region between the subunits. Although highly neglected in previous studies, this lipid shield forms a significant energy barrier of about 800k_B_T that should be overcome if two α-subunits are to dimerize. Hence, unless there is an external mechanical load that forces the monomers through the lipid pack, the lipid barrier simply hinders α-dimerization. Because, to the best of our knowledge, no mechanically effective binding of α-subunits with cytoskeletal proteins has been reported, it is unlikely for α-subunits to form homo-dimers even if they are in an active/ligand-bound state. Studies that reported α-subunit homodimerization have, in fact, observed integrins after the cell had been lysed, i.e. the plasma membrane would have been removed. We mimicked the experiments carried out by Li et al. [20] by observing the dimerization process in the absence of the plasma membrane (see Fig. 4). Our results showed that homo-oligomerization under such circumstances readily occurs. Transmembrane α-domain dissociation simulations corroborate this conclusion. Even if the homo-dimerization occurred, α-subunits would require ∼25% higher magnitude of force to dissociate than would their β-counterparts. Thus, α-subunit homo-oligomerization does not seem to be a readily available regulating event of integrin clustering. Although Wang et al. also performed homo-oligomerization and Cys scanning analysis in the absence of the cell plasma membrane, they utilized full-length integrin versus TMC domains used by Li et al [8], [20]. Insofar as integrin TMC domains are highly hydrophobic, in a hydrophilic milieu it is likely that the integrin TMC domains have bonded to hydrophobic patches on the integrin ectodomain, thereby blocking the homo-oligomerization sites on the TMC-domains. This could explain why homo-oligomerization was not observed by Wang et al.

On the other hand, transmembrane β-domains are α-helices longer than α-domains that also span the plasma membrane. They possess a large number of hydrophobic residues but the subtlety lies within a cytoplasm-proximal region of them. TMC β-domains are different from α-subunits in three distinct ways. First, TMC β-domains feature longer cytoplasmic regions that are less affected by the lipid packing phenomenon as their cytoplasmic compartment is significantly longer; second, in the resting as well as ligand-bound states β-domains maintain a 25° angle with the membrane surface normal, as opposed to α-domains that remain perpendicular to the membrane [2], [41], [42]; and finally, in β-domains the membrane-embedded region is not fully hydrophobic which gives rise to a narrowing of the lipid pack adjacent to the β-domains in the cytoplasmic leaflet of the plasma membrane. Unlike transmembrane α-domains that are more or less uniformly hydrophobic throughout their membrane-embedded length, a transmembrane β-domain length span of ∼2 nm in proximity of the cytoplasm experiences a combination of hydrophobic and hydrophilic residues that weakens the lipid packing effect around it. This region, starting from the residue D723, appears to rotate against the main body of the monomer shortly after it was equilibrated and this forms a kink that signifies this region. The rotation is caused by another hydrophobic interaction between the residue W739 and the plasma membrane. This interaction acts as a trigger for homo-dimerization. Although 1.5 nN of steering force was required to overcome the lipid pack and promote homo-oligomerization, this event could be considered physiologically feasible since it is widely established that talin head domains bind to the NPLY sequence on the β-cytoplasmic domain, a key bond that links integrins to actin filaments (Fig. 7). Other focal adhesion proteins such as α-actinin and kindlin are also capable of linking β-cytoplasmic domains to the cytoskeleton [15], [43].

Calculating the average density of the lipid bilayer around α- and β- TMC domains provides a reliable measure to compare the behavior of these two monomers when embedded in the plasma membrane (Fig. 4a and 4b). Intriguingly, the average density of lipid reaches its highest in the region between the α-domains (∼100 #/nm^3^), presenting a dense area of lipid that potentially hinders α-homo-oligomerization. Similar phenomenon occurs for β-domains except that the difference between the area enclosed by and outside the β-domains is no more than a few percents. It is noteworthy that the reported densities are averaged over five microstates of the system for each data point. Another piece of evidence was put forward by estimating the free energy changes along the reaction coordinate of β-β cytoplasmic domain homo-oligomerization. Importantly, an overall loss of free energy on the system was observed as the moving monomer approached the free one until it reached an energy bump at 2.5 nm. After the dimerization occurred, a drastic decrease in the system free energy was seen. This showed that the reaction is favorable and feasible if a source of energy exists that injects as much as 400k_B_T into the system such that it could overcome the energy barrier. As the average load sustained by an actin filament is reported to be ∼50 pN [5], an actin stretch of ∼34 nm would be sufficient to provide the amount of energy required for homo-oligomerization.

Cluzel et al. reported that activated integrin, immobilized ligands, presence of monomeric talin head domain, and phosphoinositole-4, 5-bisphosphate (PIP2) are necessary factors for integrin micro-clustering [13]. Presence of actin network, however, is not critical for *de novo*-formed integrin clusters. Along the same line, Tan et al. did not observe the linear regime of focal adhesion area growth with the force they exerted to the substrate, for nascent focal adhesions (i.e. less than 1 µm^2^ in area) [24]. Therefore, since homo-oligomerization is dependent on the presence of actins, it seems reasonable to assume neither TMC α- nor β-domains play an important role in promoting clustering in newly-formed focal adhesions. Nevertheless, TMC β-domains might be capable of mechanically regulating the clustering process when the focal adhesion is matured. Previous molecular dynamics simulations mapped out details of vinculin and α-actinin activation that is highly involved in the process of maturation [30], [31], [44], [45], [46]. An increase in the stretch level of the cytoskeleton, and particularly actin filaments that are already bonded to talin and other focal adhesion proteins and consequently to integrin β-subunits, might lead to a lateral gathering effect that brings β-subunits closer, thereby making it more probable for them to homo-oligomerize (Fig. 7b). Thus, one could hypothesize a model to link integrin activation and clustering as follows (see Fig. 7b). Individual active integrins absorb focal adhesion proteins, including talin, vinculin, etc., and eventually bind actin filaments. As the focal adhesion matures, adjacent protein hubs (∼40–50 nm apart) [47] gradually grow in size and unify. Increasing the tension in actin bundles, and consequently actin filaments that form the bundles, causes monomers to be aligned and dragged toward each other more vigorously. Homo-oligomerization interactions reinforce the binding by overcoming lipid barriers, which assembles larger numbers of integrin subunits in the focal adhesion.

Although current activation models explain how three conformations of integrin are associated with different signaling states, they fall short on elucidating alterations in the surface area of focal adhesions as the tension in focal-adhesion-binding actins grows in a 2D cell culture regime [9], [14], [15], [19], [22], [48]. Our speculated model, however, explains the phenomenon as follows. The higher the tension in an actin bundle grows, the more β-integrin homo-trimerization interactions likely occur. This in turn increases the area of the focal adhesion in a 2D culture, which implies that a larger number of active integrins are recruited if an elevated magnitude of force is to be borne by the corresponding actin bundle. The extended ectodomain of integrin and presence of a hinge-like region between the ectodomain and TMC domains enables integrin β-cytoplasmic tails to homo-oligomerize while their ectodomains are bonded to adjacent ECM ligands. The GXXXG sequences are binding sites for hetero-trimerization. As long as the cytoskeletal tension is present and integrins are stabilized in a homo-trimeric interaction, they are not expected to return to their resting state, where α- and β-domains are bonded at their IMCs and OMCs.

In summary, our simulations showed that TMC α-domains are unlikely to contribute to the process of integrin clustering. However, TMC β-subunits' geometry as well as composition make them an important candidate to drive the integrin clustering process. Previous experiments implicitly neglected energetic effects of the plasma membrane, obtaining apparently contradictory outcomes with lysed cells. Hence, we suggest future experimental studies of the field focus on clustering of integrin β-subunits over the course of the focal adhesion formation and collapse either *in vivo* or within artificial lipid bilayers. Furthermore, in order to obtain a thorough understanding of the focal adhesion growth, effects of other environmental factors, such as extracellular and cytoplasmic ion concentration, on integrin clustering should be examined.

## Computational Procedures

### Normal mode analysis

Integrin αllbβ3 ectodomain (PDB ID: 3FCS chains A and B [49]) and transmembrane-cytoplasmic domains (PDB ID: 2KNC [50]) were downloaded from the Protein Data Bank (PDB) and combined to build the full-length integrin molecule, assuming a covalent bond between residues Cys959 and Gly955 in the α-subunit, and Gly684 and Gly690 in the β-subunit. The natural vibration frequencies of the full-length integrin molecule were determined using the normal mode analysis (NMA) software WEBnm@ [51]. The normal mode matrix, which is a function of integrin molecular structure, shows natural movements in flexible molecular regions and little movement in rigid regions. WEBnm@ uses the MMTK [52] software internally and computes natural frequencies using Hinsen's computational methods [53], which calculates approximate normal modes by determining the eigenvectors of the matrix of second derivatives of potential energy with respect to displacement of the Cα atoms of each residue. The potential energy function used for this calculation utilizes a Hookian potential between Cα atoms within an 8 Å cutoff distance. Because NMA represents movements resulting from the overall structure, the use of Cα atoms is sufficient for NMA calculations [54].

### Molecular dynamics

All molecular dynamics simulations are carried out with the program NAMD2.7 [36], using the CHARMM27 force field for lipids and CHARMM22 for proteins [55]. The transmembrane-cytoplasmic domains of the integrin αllbβ3 molecule are taken from Protein Data Bank (PDB ID: 2KNC). Integrin subunits are embedded in a 1-palmitoyl-2-oleoyl-sn-glycero-3-phosphocholine (POPC) lipid bilayer, using the software VMD [56]. In all simulations, overlapping lipid chains are removed afterward and the membrane is solvated in an explicit water box with ionic NaCl concentration of 8 mM [57]. In all simulations integrin is assumed to be in an activated state as transmembrane-cytoplasmic domains of α- and β-subunits are apart. The temperature and pressure of the system are held constant at 1atm and 310K, using Langevin's piston and Hoover's method [36]. Coordinates, energy, and steering forces were recorded every 1 ps or 10 ps and the time step was 2 fs. The cutoff distance for non-bonded interactions was 1.2 nm. For all simulations, particle mesh Ewald (PME) method was used for electrostatic force calculations [36]. The cutoff distance for non-bonded interactions was 1.2 nm. The hydrogen atom bond length was constrained using SHAKE method. SHAKE method fixes bond lengths between large atoms and hydrogen atoms, preventing unnecessary calculation of irrelevant interactions [58].

In α-α and β-β homo-oligomerization simulations, the two monomers were initially positioned 5 nm apart within the lipid bilayer and the system was solvated and ions were randomly added to the system afterward. Subsequently, the system was minimized for 4 ps and equilibrated for 0.5 ns. The results were visualized using VMD [56]. The steered α-monomer was pushed at Cα of residues Ala956, Gly972, Ala 986, and Gly1007, whereas β-monomer is forced at Cα of residues Val695, Gly708, Ala728, and Ala742 with a constant velocity of either 0.025 Å/ps or 0.0025 Å/ps. The other monomer is either fixed at the same atoms or free to move.

To observe the α-dimerization interaction, two α-dimers are placed initially 2 nm apart and equilibrated for 1 ns. After bonds are formed and the system reached equilibrium, one monomer is constrained at the same four atoms while the other is pulled on from the atoms corresponding to the constrained one, away from the fixed monomer. β-trimerization is scrutinized by primarily placing three transmembrane-cytoplasmic β-domains on vertices of an equiangular triangle each 2 nm away from its adjacent monomers. The system is then equilibrated for 1 ns until the trimerization interaction assumes an equilibrium state. Then, one monomer is pulled while others are constrained. This was continued until the steered monomer was completely separated from its neighboring β-monomers. All dimerization and trimerization simulations were equilibrated for 1.5 ns to confirm the stability of interactions.

### Plasma membrane density calculations

The density of the cytoplasmic compartments of lipid chains were measured for the entire length of the plasma membrane along the line that connected the two subunits embedded in the membrane. A 100×50 Å and a 100×150 Å lipid patch were used to embed α- and β-domains in, respectively. The lipid patches and integrin subunits were minimized and equilibrated. Nine (each one 100 ps after the previous one) and five (each one 500 ps after the previous one) snapshots of the system in equilibrium were averaged for α- and β-domains, respectively.

### Free energy calculations

In order to calculate the free energy profile of the α-α and β-β homo-oligomerization of cytoplasmic domains, we invoked Jarzynski method [33]. This method is a powerful tool for calculating free energy profiles along reaction coordinates. It is applicable to non-equilibrium processes on the energy landscape of the system. The method employs the following formula to relate the free energy change of the system to the external work done on the system:
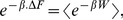
(4)where *β*, *Δ*F, and *W* are *1/k_B_T*, change in free energy, and external work, respectively. Provided the number of samplings is extremely high, the formula applies independent of the process speed. Nonetheless, reasonable approximations have been made using Jarzynski method for steered molecular dynamics, when the system has been sampled only 10 times. Therefore, we minimized and equilibrated the system 10 times including two integrin TMC subunits, 5 nm apart, embedded in a patch of plasma membrane. Afterwards, one subunit was steered toward the other from its cytoplasmic tail, while the other subunit was left unconstrained. The identical process of steering was repeated once starting from each of the 10 equilibrated systems. In order to avoid the bias toward the samples with smaller numbers, the free energy was calculated using the second order expansion of Eq. 4, as follows:
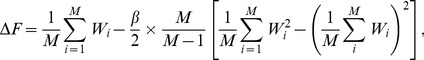
(5)where *M* and *W_i_* are number of samples and the work corresponding to the *i-th* sample.

## Supporting Information

Figure S1 **The moving pattern of the crystal structure of integrin in deformation mode 7, which is the mode with highest contribution to the deformation pattern of the protein, at the two deformation extremes (right and left).** It can be seen that the transmembrane-cytoplasmic domain rotates past the extracellular domains almost without introducing any significant conformational change to the extracellular domain. The blue and red dashed lines show the paths taken by β- and α-subunits when vibrating at mode 7.
(TIF)

Figure S2 **Related to** **Figure 1** **; Modal deformation energy increases rapidly with the mode number, making higher modes have much smaller contribution to the protein deformation.**
(TIF)

Figure S3 **Related to** **Figure 4** **; Some minor interactions occur when one integrin α-subunit is dragged toward the other.** (a) Interaction energy between the two monomers does not show any significant changes as the two monomers approach one another. Positive energy magnitudes are indicative of interactions between hydrophobic groups of the monomers that fall outside the plasma membrane. The steering force and interaction energies all correspond to the slow steering rate. In presence of the GAMG sequence there are a number of energy spikes whereas almost no interaction is observed in the absences of the GAMG sequence. (b) Upper parts of each α-monomer are chopped off in order to illustrate the interaction site more clearly. The two α-monomers are depicted when they have already reached their final distance. Hydrophobic interactions of the GAMG sequence (Gly: blue, Met: purple, and Ala: green) with a hydrophobic region (yellow) of the free monomer is illustrated.
(TIF)

Figure S4 **A lipid pack forms around the transmembrane domain which moves along with the monomer.** (a) Lipid atoms initially located within 10 Å of the α-monomer are visualized and monitored over the course of the simulation. Although some lipid chains are dispersed away as the monomer moves along, the major part of the lipid pack compartment remains attached to the monomer. (b) Shows the distance between the monomer's center of mass and three lipid chains located at different distances at the rear side of the moving monomer along the line that connects the two monomers. The graph clearly shows that lipid chains that are closer to the monomer move along with the monomer, whereas, the ones that are initially more distant fall behind the moving monomer more rapidly.
(TIF)
