# Supplementary figures and images for: Localized Lipid Packing of Transmembrane Domains Impedes Integrin Clustering
Source: PLoS Comput Biol. 2013 Mar 14;9(3):e1002948. doi: 10.1371/journal.pcbi.1002948 (PMC3597534; doi:10.1371/journal.pcbi.1002948)

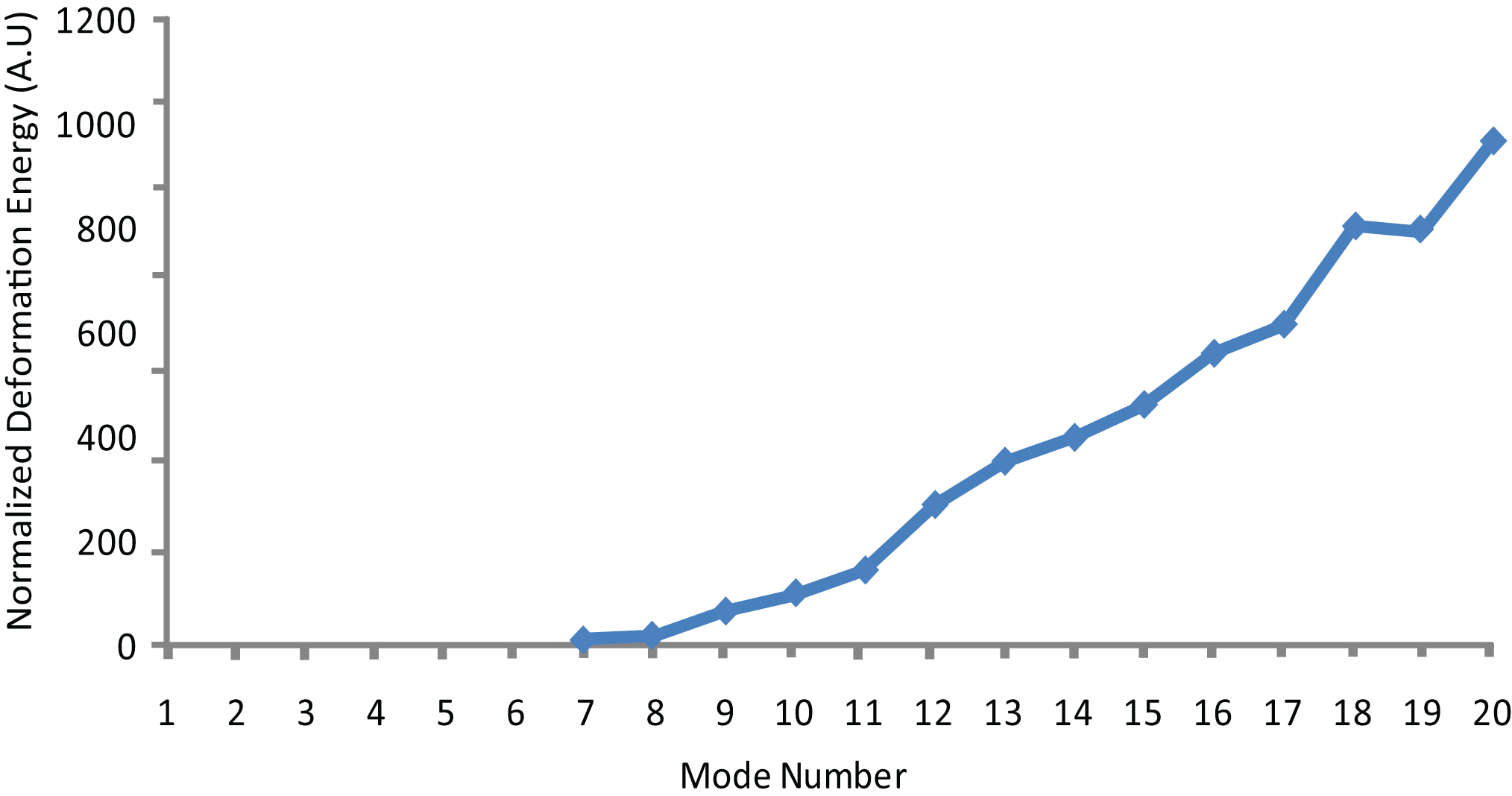

Supplement: Figure S1 — The moving pattern of the crystal structure of integrin in deformation mode 7, which is the mode with highest contribution to the deformation pattern of the protein, at the two deformation extremes (right and left). It can be seen that the transmembrane-cytoplasmic domain rotates past the extracellular domains almost without introducing any significant conformational change to the extracellular domain. The blue and red dashed lines show the paths taken by β- and α-subunits when vibrating at mode 7. (TIF) [file pcbi.1002948.s001.tif]

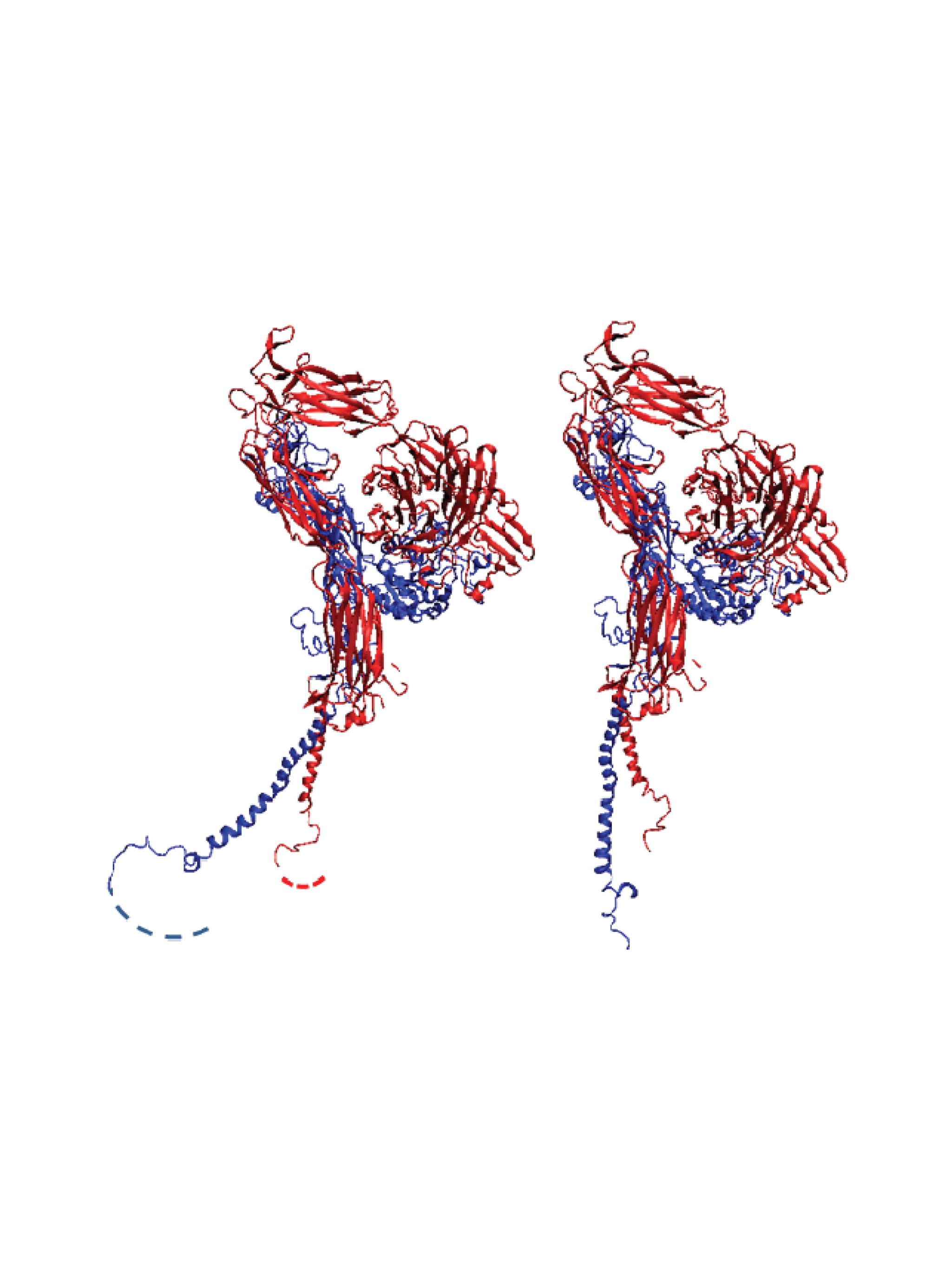

Supplement: Figure S2 — Related to Figure 1 ; Modal deformation energy increases rapidly with the mode number, making higher modes have much smaller contribution to the protein deformation. (TIF) [file pcbi.1002948.s002.tif]

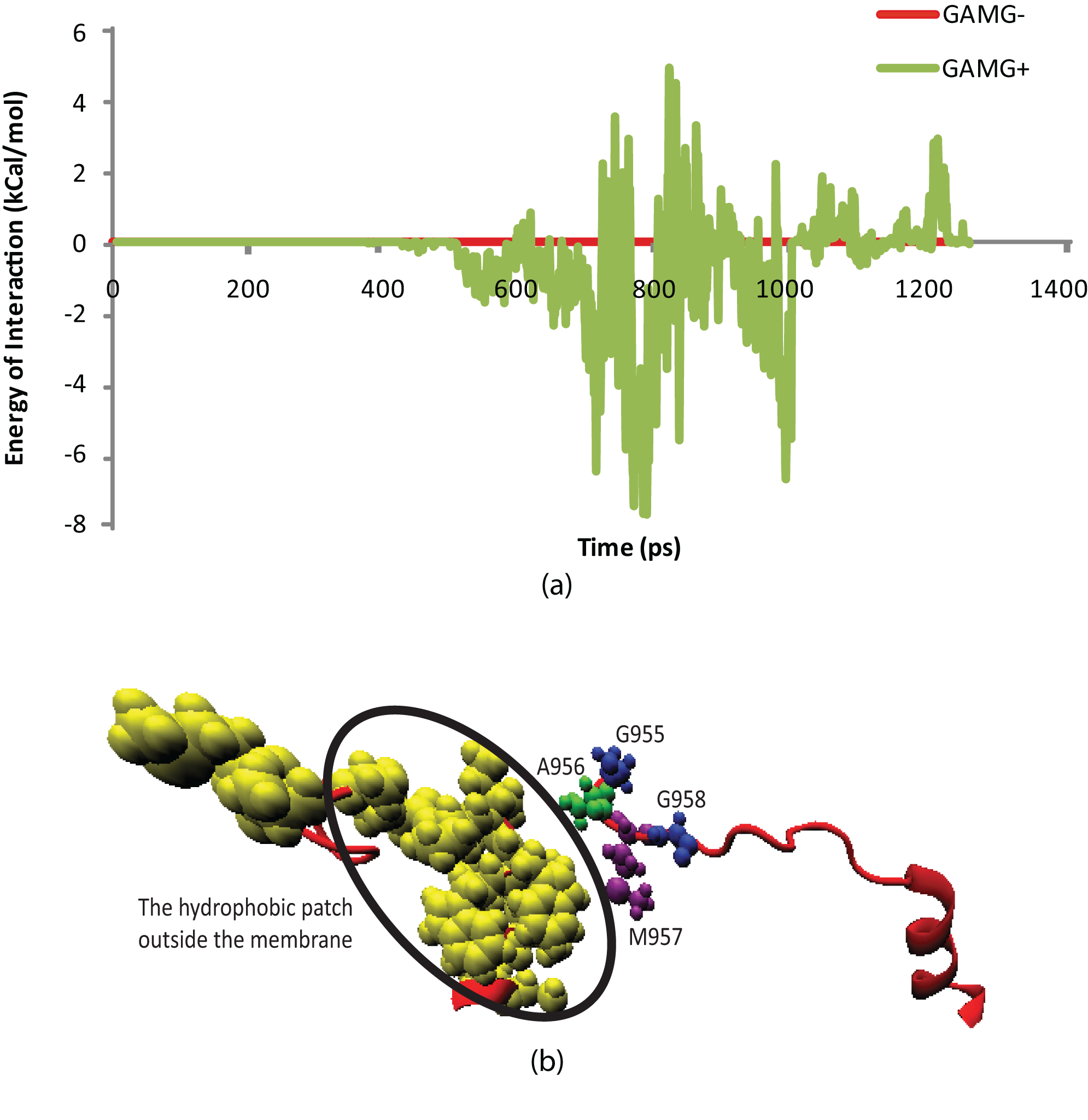

Supplement: Figure S3 — Related to Figure 4 ; Some minor interactions occur when one integrin α-subunit is dragged toward the other. (a) Interaction energy between the two monomers does not show any significant changes as the two monomers approach one another. Positive energy magnitudes are indicative of interactions between hydrophobic groups of the monomers that fall outside the plasma membrane. The steering force and interaction energies all correspond to the slow steering rate. In presence of the GAMG sequence there are a number of energy spikes whereas almost no interaction is observed in the absences of the GAMG sequence. (b) Upper parts of each α-monomer are chopped off in order to illustrate the interaction site more clearly. The two α-monomers are depicted when they have already reached their final distance. Hydrophobic interactions of the GAMG sequence (Gly: blue, Met: purple, and Ala: green) with a hydrophobic region (yellow) of the free monomer is illustrated. (TIF) [file pcbi.1002948.s003.tif]

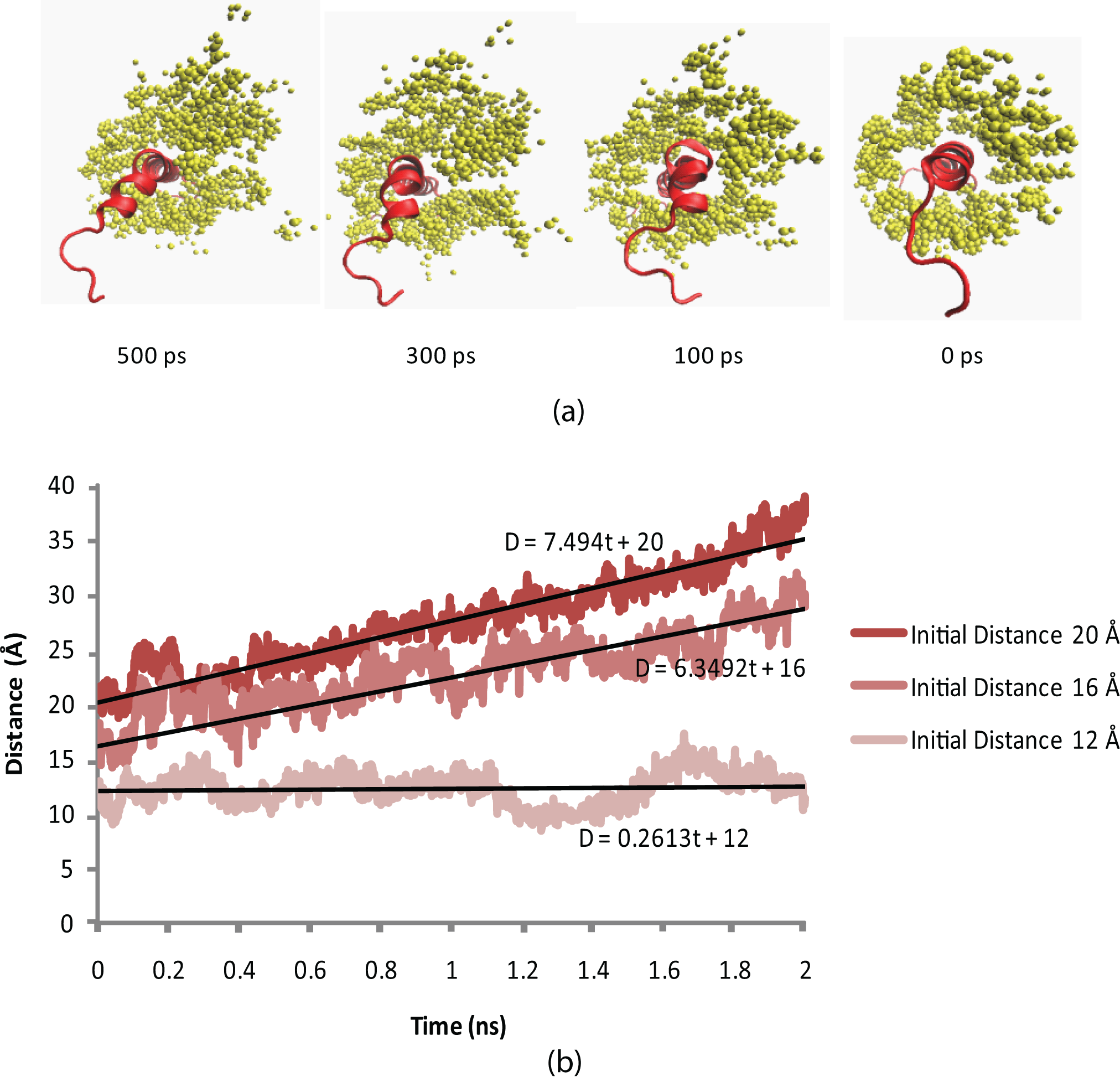

Supplement: Figure S4 — A lipid pack forms around the transmembrane domain which moves along with the monomer. (a) Lipid atoms initially located within 10 Å of the α-monomer are visualized and monitored over the course of the simulation. Although some lipid chains are dispersed away as the monomer moves along, the major part of the lipid pack compartment remains attached to the monomer. (b) Shows the distance between the monomer's center of mass and three lipid chains located at different distances at the rear side of the moving monomer along the line that connects the two monomers. The graph clearly shows that lipid chains that are closer to the monomer move along with the monomer, whereas, the ones that are initially more distant fall behind the moving monomer more rapidly. (TIF) [file pcbi.1002948.s004.tif]
